# Neural Correlates of the Perception of Spoiled Food Stimuli

**DOI:** 10.3389/fnhum.2016.00302

**Published:** 2016-06-21

**Authors:** Christoph A. Becker, Tobias Flaisch, Britta Renner, Harald T. Schupp

**Affiliations:** Department of Psychology, University of KonstanzKonstanz, Germany

**Keywords:** eating, disgust, fMRI, ERP, LPP

## Abstract

The elicitation of disgust by the view of spoiled and rotten foods is considered as an adaptation preventing the ingestion of harmful microorganisms and pathogens. To provide an effective behavioral defense, inedible food items need to be detected automatically, i.e., in the absence of explicit processing goals, early in the processing stream, and triggering an alarm response, i.e., increased attentional capture. To examine these hypotheses, a set of stimulus material consisting of images of perishable foods (i.e., dairies, meats, fruits, and vegetables) at various stages of natural decay ranging from appetitive to disgusting was developed. In separate sessions, functional imaging and dense sensor event related potential (ERP) data were collected while participants (*N* = 24) viewed the stimulus materials. Functional imaging data indicated larger activations in the extrastriate visual cortex during the processing of inedible as compared to edible food items. Furthermore, ERP recordings indicated that the processing of inedible food stimuli was associated with a relative positivity over inferior occipital sensor sites already at early stages of processing (<200 ms), and subsequently, an increased late positive potential (LPP) over parieto-occipital sensor regions. Taken together, these findings demonstrate the brain’s sensitivity to visual cues of foods that are spoiled or rotten.

Food is an essential source of energy and necessary for survival. Accordingly, the sight of potential foods can arouse a strong motivation for ingestion. However, natural decay can turn even the most appetizing foods into a dangerous substance. Throughout most of humans’ history, discrimination of edible and inedible food for pathogen avoidance was of primal importance (Cockburn, 1971; Wolfe et al., 2007). However, the threat of ingesting pathogens is still present in modern times, even in the USA (CDC, 2014). In the present research, natural decay of foods was used to investigate neural processes associated with the discrimination of edible and inedible food items.

For omnivores species, such as humans, the ability to discriminate between edible and inedible foods is only partly based on innate mechanisms. Experiences and food enculturation across the lifespan also significantly shape responses to food items (Rozin and Vollmecke, 1986). Specifically, food items may be selected or rejected according to sensory-affective characteristics, attitude and beliefs, or symbolic meaning and ideational factors (Fallon et al., 1984; Rozin and Fallon, 1987; Steptoe and Wardle, 1999; Renner et al., 2012).

Among the factors relevant for the selection of foods, previous research primarily focused on the processing of calorie-dense foods. Functional imaging studies indicate that high-calorie items activate visual-associative cortical brain regions to a greater degree as low-calorie control items and, albeit much less consistent, elicit greater activation in brain regions implicated in motivational processes, i.e., insula, cingulate cortex, amygdala, ventral striatum, and orbitofrontal cortex (Killgore et al., 2003; Beaver et al., 2006; Cornier et al., 2007; Goldstone et al., 2009; Passamonti et al., 2009; Born et al., 2011).

Event related potential (ERP) studies further strengthened the notion that selected food stimuli modulate attention. For instance, the comparison of high- and low-fat and liked and disliked foods (Toepel et al., 2009; Harris et al., 2011, 2013), and vegetarians’ and omnivores’ processing of meat dishes (Stockburger et al., 2009) indicated increased late positive potentials (LPP) between 300 and 700 ms. In addition, ERP studies also showed that low- and high-fat foods are discriminated early in the processing stream, i.e., ~150–200 ms (Toepel et al., 2009; Blechert et al., 2011; Harris et al., 2011, 2013; Meule et al., 2013). Overall, there is accumulating evidence by functional imaging and ERP studies that the brain selectively responds to the appetitive value and significance of food stimuli.

Little attention has been paid in previous research to the specific factors associated with the rejection of foods. A large array of functional imaging studies showed that disgust-eliciting stimuli capture attention. However, these studies usually included a broad range of disgust elicitors (e.g., Schienle et al., 2006; Stark et al., 2007) or used different food items in the disgust and control condition (Beaver et al., 2006; Calder et al., 2007). Overall, the resolution was not sufficient in previous research to reveal specific neural correlates associated with discriminating inedible from edible food items.

The robust elicitation of disgust by the view of spoiled and rotten foods is seen as adaptation to prevent the ingestion of pathogens (Rozin and Fallon, 1987; Oaten et al., 2009; Schaller and Park, 2011; Tybur et al., 2013; Rozin et al., in press). To provide an effective behavioral defense, inedible foods need to be detected automatically and trigger an alarm response, i.e., increase attentional capture. To examine this hypothesis, an effort was made to develop a set of stimulus material consisting of images of perishable foods (see Foroni et al., 2013) at various stages of natural decay (see Figure 1) and collect functional imaging and dense sensor ERP data while participants viewed the stimulus materials. With regard to functional magnetic resonance imaging (fMRI), the main prediction was that contaminated food stimuli show increased blood oxygen level-dependent (BOLD) signal activations in extrastriate visual cortex. Furthermore, regarding ERPs, it was predicted that brain waves to edible and inedible food stimuli would differ at early stages of processing, i.e., 150–200 ms, and that inedible food stimuli elicit a larger LPP than edible foods.

**Figure 1:**
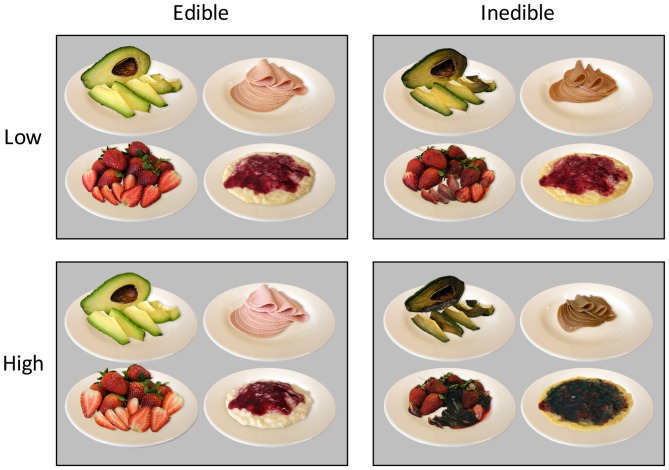
**Representative example stimuli showing the natural decay of food items in four stages**.

## Materials and Methods

### Participants

Twenty-four participants (12 females, 3 left-handed) between 21 and 35 years of age (*M* = 24.4, *SD* = 3.45) with normal or corrected-to-normal vision participated in the study. Participant’s body mass index (BMI) ranged between 19.7 and 27.6 (*M* = 22.9, *SD* = 2.64) for females and between 20.2 and 28.1 (*M* = 23.2, *SD* = 2.31) for males. Furthermore, the German adaptation of the Restraint Scale (FEV) served to assess restrained eating (Heatherton et al., 1988; Pudel and Westenhöfer, 1989). Male and female participants (*M* = 3.7 and 7.4, *SD* = 3.03 and 6.0, respectively) scores were similar on the FEV-Restraint Scale (range: 1–21), *t*_(22)_ = −1.9, ns. With regard to diet, three participants reported that they do not consume any meat products^1^. Participants were recruited at the University of Konstanz and received either course credit or €8 per hour. The study was approved by the ethical review board of the University of Konstanz and informed consent was acquired from all participants. Participants were not included in the study when they had a history or currently suffered from psychiatric, neurological, or endocrine diseases or taking medication that affects the endocrine or central nervous system.

### Stimulus Material

According to the main aim of the study, a food picture database was developed containing stimuli showing the natural decay of food items, i.e., from appetitive to disgust (see Figure 1). In a first step, food categories were selected according to the following criteria: first, the food items should reveal a continuous process of natural decay. Second, the food items should be common in the German diet, minimizing effects due to unfamiliarity with the respective foods. Third, each food category should contain several exemplars in order to vary the items within a food category. Based on these criteria, fruits, vegetables, dairy products, and meats were selected as food categories. After selecting appropriate food items, the most appetitive stimulus was created by choosing a picture composition in which the food item appeared highly appetitive and ready to eat. The same picture composition was used throughout the natural decay transition phase. Specifically, over the course of days and weeks, images of the food items were repeatedly taken until the food item became disgusting. To increase the quality of the food database, image processing and editing software were utilized to standardize the stimulus materials. In particular, for each image, the food product was released from the background, normalized in shape and size to the respective initial food, placed on an empty white plate, equipped with an artificial shadow, and sharpened to increase perceptibility. Each image contained a colored display of the respective food, sized at 640 × 480 pixels with a resolution of 72 pixels per inch, and an 8 bit RGB color resolution. Overall, the food stimulus database contained pictures from four food categories, five food items per category, and, for each food item, a series of 10–15 pictures to capture natural decay.

A first pilot study (*N* = 9) was conducted to select the stimulus materials for the present study. Participants were asked to choose the four images of the stream of pictures available for each food item capturing the continuum of natural decay, resulting in 20 images each for the high edible, low edible, low inedible, and high inedible condition. Specifically, the edible food category was represented by the most appetitive and a less appetitive but edible food stimulus while the inedible category consisted of heavily and slightly disgusting exemplars. A second pilot study with independent raters (*N* = 9) evaluated each of the chosen pictures on dimensions of edibility and pleasantness (9-point Likert scales). Ratings of edibility and pleasantness were highly correlated, *r* = 0.98, *p* < 0.001. Analysis of variance (ANOVA) analysis containing the factors Edibility (inedible vs. edible), and Intensity (low vs. high) revealed highly significant differences among the edible and inedible food categories (*p* < 0.001) as well as interacting effects with low and high intensity (*p* < 0.001) for pleasantness and edibility ratings.

### Procedure

An initial screening session served to inform participants about the study and to check their eligibility for study participation. Participants were examined in two sessions in which either fMRI or dense sensor EEG data were collected. The order of these sessions was counter-balanced and 1 week apart. To control for variations in circadian rhythm, testing occurred at 6 pm. Instructions for both sessions required participants to follow their normal eating and drinking habits and to intake their last meal 2 h before scanning. Except for technical issues of the respective imaging method, the procedure of the experimental protocol was identical across both sessions.

During both sessions, participants came to the laboratory and were checked for possible risks in relation to magnetic resonance imaging/electroencephalograph (MRI/EEG) acquisition and gave their informed consent. Immediately before entering the MRI/EEG recording room, participants provided ratings of hunger and thirst. During MR acquisition, participants were situated head first in a supine position inside the scanner. Experimental presentation was realized with a visual system (NordicNeuroLab, Bergen, Norway) positioned in front of the participant’s eyes. During EEG acquisition, participants were situated inside a comfortable chair and viewed the pictures on a 22-inch PC monitor at horizontal and vertical visual angles of 13.9° and 10.5°, respectively. In both sessions, participants were informed about the presentation of pictures and instructed that s/he should attend to each picture the entire time it appeared on the screen. MRI/EEG sessions used the same event-related paradigm. Specifically, pictures were shown for 2 s, followed by a variable inter-stimulus interval (ISI) showing a white fixation cross on a black background. The ISI was exponentially distributed with a mean of 3 s and a range of 2–5 s (see for example Amaro and Barker, 2006). The stimulus set (*N* = 80) was repeated three times with no more than two repetitions of the same category allowed, resulting in 240 trials overall. In the fMRI session, a T1 weighted structural scan was obtained following functional imaging. Subsequently, outside the recording room, a second rating of hunger and thirst was obtained, and participants additionally rated all pictures on pleasantness as well as on emotional dimensions of valence and arousal. After the second session, participants filled out a questionnaire including the Restraint Scale (Pudel and Westenhöfer, 1989) and the Eating Motivation Survey (TEMS; Renner et al., 2012). Afterwards, participants were given reimbursement or course credits, debriefed, and thanked for study participation.

### Stimulus Ratings

Participants used vertical bars to rate the pleasantness of the stimuli on a 9-point Likert scale, ranging from unappetizing over neutral to appetizing. Furthermore, the Self-Assessment Manikin were used to rate the stimuli on emotional dimensions of valence and arousal on a 9-point Likert scale, ranging from unpleasant over neutral to pleasant for valence and from calm to exciting for arousal (Bradley and Lang, 1994).

**Figure 2:**
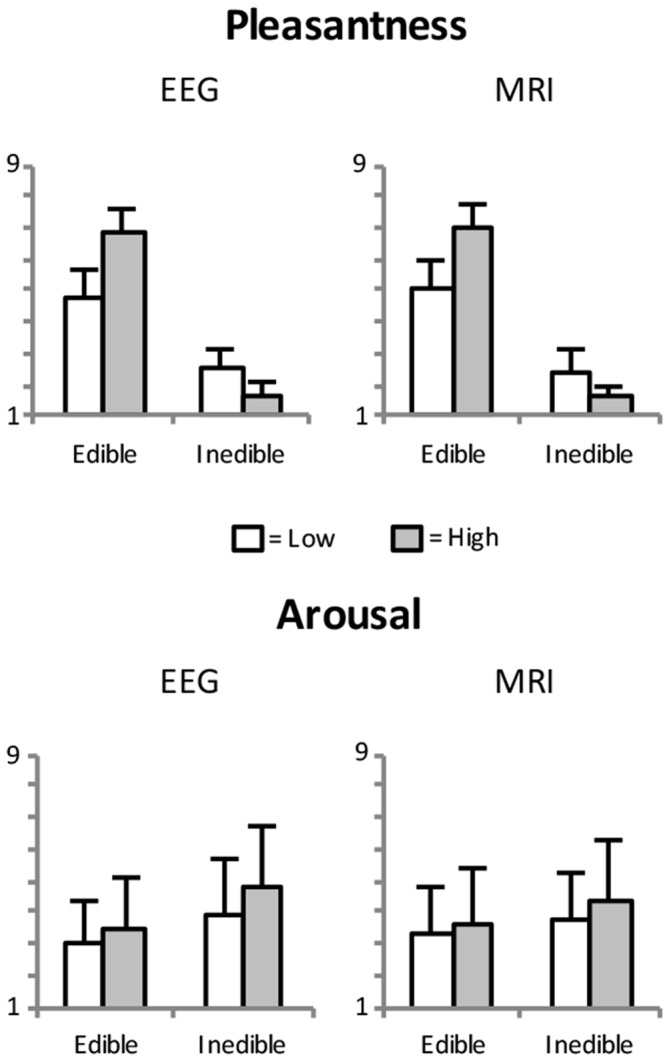
**Mean pleasantness and arousal ratings as a function of edibility and intensity of the food stimuli.** Error bars indicate standard deviations.

As expected, pleasantness and valence ratings showed a strong positive relationship, *r* = 0.99, *p* < 0.001. For brevity, only pleasantness ratings are reported here. Furthermore, initial analysis revealed similar pleasantness and arousal ratings for both, EEG and fMRI sessions (see Figure 2). All analyses were initially conducted including the factor *Session* but no significant main effects or interactions were found involving this factor.

### Hunger and Thirst Ratings

Hunger and thirst ratings were collected using a 7-point Likert scale, ranging from not hungry (not thirsty) to very hungry (very thirsty), respectively. Rating data were obtained immediately before and at the end of MRI/EEG acquisition. Hunger and thirst ratings were entered into two-way repeated measures ANOVAs with the factors *Time of record* (pre vs. post) and *Session* (EEG vs. MRI).

In accordance with our eating instructions, participants did not show altered hunger ratings across the experiment (Pre: *M* = 3.8, *SD* = 1.45; Post: *M* = 4.0, *SD* = 1.48) nor between sessions (EEG: *M* = 3.7, *SD* = 1.63; MRI: *M* = 4.0, *SD* = 1.51), *F*s_(1,23)_ < 2.9, ns. However, the participants were significantly more thirsty at the end of the respective experiment (Pre: *M* = 4.0, *SD* = 1.01; Post: *M* = 4.6, *SD* = 1.12), *F*_(1,23)_ = 17.2, *p* < 0.001, partial *η*^2^ = 0.43, but not between sessions (EEG: *M* = 4.4, *SD* = 1.31; MRI: *M* = 4.2, *SD* = 1.17), *F*_(1,23)_ = 0.4, ns.

### MRI Data Acquisition and Analysis

MR acquisition took place on a 1.5 T Philips Intera MR system (Philips, Hamburg, Germany). In a single functional scanning session, 482 volumes of a T2* weighted Fast Field Echo, Echo Planar Imaging sequence utilizing parallel scanning technique were acquired (SENSE; Pruessmann et al., 1999). In plane resolution was 3 × 3 mm and slice thickness was 3.5 mm (32 axial slices; no gap; FOV = 240 mm; acquisition matrix = 80 × 80; *TE* = 40 ms; flip angle = 90°; *TR* = 2500 ms). In addition, a standard T1 weighted high resolution structural scan with 1 × 1 × 1 mm voxel resolution was obtained.

Preprocessing and statistical analysis of the functional data was conducted using SPM8 (Wellcome Department of Imaging Neuroscience, University College London, UK; Friston et al., 1994). Preprocessing steps included realignment and slice time correction for the functional images. No participant displayed head movements exceeding 3 mm or 3° on any axis. Images were normalized to the MNI EPI template and resampled at 3 × 3 × 3 mm voxel size. A Gaussian spatial kernel of full width at half maximum (FWHM) with an 8 mm radius was used for smoothing the data.

Single subject data were modeled with one session, containing four covariates of interest representing the food picture onsets for each condition as well as covariates of no interest, including six movement parameters and one covariate incorporating an overall intercept to the model. Group level random effects analysis combined all subjects’ covariates of interest into a model with the within factors *Edibility* (inedible vs. edible) and *Intensity* (low vs. high) and the between factor *Session* (MRI-EEG vs. EEG-MRI). Linear contrasts were computed for the main effects of *Edibility* and *Intensity* as well as for the interaction of *Edibility by Intensity*. To control for possible session effects, all linear contrasts were exclusively masked by an *F*-contrast of *Session* at *p* < 0.05 (uncorrected), discarding all voxels showing a main effect of *Session*. However, a separate stream of analyses without controlling for session order effects revealed no further effects beyond those reported in the results section. Activations of the resulting SPM(t) maps were considered meaningful if they reached an uncorrected threshold of *p* < 0.001 at the voxel level and a cluster-level threshold of *p* < 0.05, corrected for multiple comparisons (Family-wise error, FWE).

### EEG Data Acquisition and Analysis

Electrophysiological data were collected using a 257-lead Hydro-Cell Geodesic Sensor Net (EGI: Electrical Geodesics, Inc., Eugene, OR, USA). The EEG was recorded continuously with a sampling rate of 250 Hz, with the vertex sensor as reference electrode, and online filtered from 0.1 to 100 Hz using Netstation acquisition software and EGI amplifiers. Impedances were kept below 50 kΩ, as recommended for this type of amplifier. Electromagnetic Encephalography Software (EMEGS; Peyk et al., 2011) was used for analysis. Data editing and artifact rejection were based on a method for the statistical control of artifacts specifically devised for analyzing dense sensor EEG recordings (Junghöfer et al., 2000). Preprocessing steps included 40 Hz digital low-pass filtering, epoching from −200 to 1000 ms, artifact detection, ocular artifact correction using a correlative eye movement algorithm (Schlögl et al., 2007), and bad sensor interpolation. On average, ERP waveforms were based on a trial number of 50.1 trials (*SD* = 5.0), which did not differ between the four stimulus categories, *F*_(3,92)_ = 0.3, ns. Finally, the data were converted to an average reference and baseline-adjusted for pre-stimulus (100 ms) ERP activity.

Single sensor waveform analyses were used to determine statistically significant effects. Specifically, data were low-pass filtered (15 Hz) and each time point and sensor was submitted separately to a repeated measure ANOVA including the within factors *Edibility* (Inedible vs. Edible) and *Intensity* (Low vs. High). Initial analyses included also the between factor *Session* (EEG-MRI vs. MRI-EEG). Again, there were no significant findings involving this factor, which was consequently dropped from further consideration. To account for the multiple comparisons problem, a cluster-based permutation test with *N* = 1000 permutations was performed (Maris and Oostenveld, 2007). Sensor clusters were considered meaningful if they reached a single-sensor inclusion threshold of *p* < 0.01 and a cluster-level threshold of *p* < 0.05, corrected for multiple comparisons.

## Results

### Stimulus Ratings

Pleasantness ratings confirmed the *a priori* categorization of the stimulus materials. As shown in Figure 1, there was the expected difference between edible and inedible food stimuli, *F*_(1,23)_ = 890.3, *p* < 0.001, partial *η*^2^ = 0.98. Furthermore, as indicated by the interaction of Edibility by Intensity, *F*_(1,23)_ = 571.3, *p* < 0.001, partial *η*^2^ = 0.96, the high disgust category was perceived as more unpleasant than the low disgust category, *t*_(23)_ = −11.2, *p* < 0.001, *d* = 4.67, and the high appetitive category as more pleasant than the low appetitive category, *t*_(23)_ = 13.6, *p* < 0.001, *d* = 5.65.

Arousal ratings revealed main effects of *Edibility*, *F*_(1,23)_ = 9.5, *p* < 0.005, partial *η*^2^ = 0.29, and *Intensity*, *F*_(1,23)_ = 29.3, *p* < 0.001, partial *η*^2^ = 0.56. Specifically, as shown in Figure 2, inedible food stimuli are perceived as more arousing than edible food stimuli and salient food pictures (high appetite and disgust) were rated as more arousing than less salient food pictures.

### fMRI Data

#### Edibility: Main Effects and Interactions

**Figure 3:**
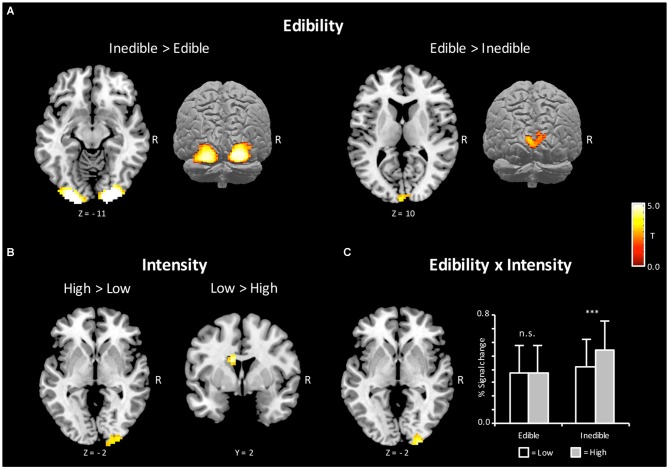
**Brain sections and cortical projections illustrating blood oxygen level-dependent (BOLD) activations during the magnetic resonance imaging (MRI) session. (A)** Main effect of Edibility. Left: the bilateral extra striate visual cortex showed a preferential coding for inedible foods. Right: the left primary visual cortex showed a preferential coding for edible foods. **(B)** Main effect of Intensity. Left: the right extra striate cortex showed a preferential coding for extreme food categories. Right: the left caudate showed a preferential coding for mild food categories. **(C)** Left: illustration of the interaction *Incentive by Intensity* in the right extra striate cortex. Right: extracted percent signal changes for the cluster on the left (Mean and SD). For illustrative purposes, *post hoc* tests between conditions were calculated to reveal the nature of interaction. Statistical maps are thresholded with a single-voxel inclusion of *p* < 0.001 and a cluster-level threshold of *p* < 0.05 family-wise error (FWE).

According to the hypothesis of a behavioral defense mechanism, a first stream of analysis served to determine brain regions, which showed increased BOLD activity to inedible when compared with edible food items [Inedible > Edible]. As shown in Figure 3, bilateral regions of the extra striate visual cortex (*x* = 21, *y* = −100, *z* = −5, Size = 367, PeakZ = 7.05 and *x* = −21, *y* = −103, *z* = −11, Size = 335, PeakZ = 6.96) revealed increased BOLD activation during the processing of inedible as compared to edible food stimuli. In a second step, brain regions were determined, which showed larger BOLD activity to edible when compared with inedible food stimuli. The contrast [Edible > Inedible] revealed an increased BOLD activation in the left primary visual cortex (*x* = −6, *y* = −97, *z* = 16, Size = 69, PeakZ = 4.36).

According to a motivational pathogen-avoidance hypothesis, the processing of inedible food stimuli should be accentuated for the extreme stimulus category. To test for this assumption, the cross interaction contrast [(Inedible_High_ > Edible_High_) > (Inedible_Low_ > Edible_Low_)] was computed and masked inclusively with the contrast of [Inedible > Edible] (*p* < 0.05, uncorrected). A region of the right extra striate cortex (*x* = 27, *y* = −100, *z* = −2, Size = 51, PeakZ = 4.24), which had shown preferential coding for natural decayed food, emerged from this contrast. As illustrated in Figure 3C, this brain region was primarily sensitive to the high disgust category. Furthermore, the reverse interaction contrast [(Edible_High_ > Inedible_High_) > (Edible_Low_ > Inedible_Low_)], masked inclusively with the contrast of [Edible > Inedible], revealed no significant effects, i.e., no brain region showed increased activation to high appetitive foods.

Of note, the unsigned cross interaction contrasts did not reveal additional regions.

#### Main Effect of Intensity

Further analysis determined effects of intensity independent on behavior orientation. The contrast [High > Low] intensity revealed a region in the extrastriate cortex (*x* = 21, *y* = −103, *z* = 1, Size = 94, PeakZ = 3.79) overlapping with the increased BOLD activations observed for inedible foods. The reverse contrast [Low > High] intensity showed an enhanced BOLD response in the left caudate region (*x* = −12, *y* = 2, *z* = 22, Size = 49, PeakZ = 4.70; see Figure 3B).

### Event-Related Potentials

#### Edibility: Main Effects and Interactions

**Figure 4:**
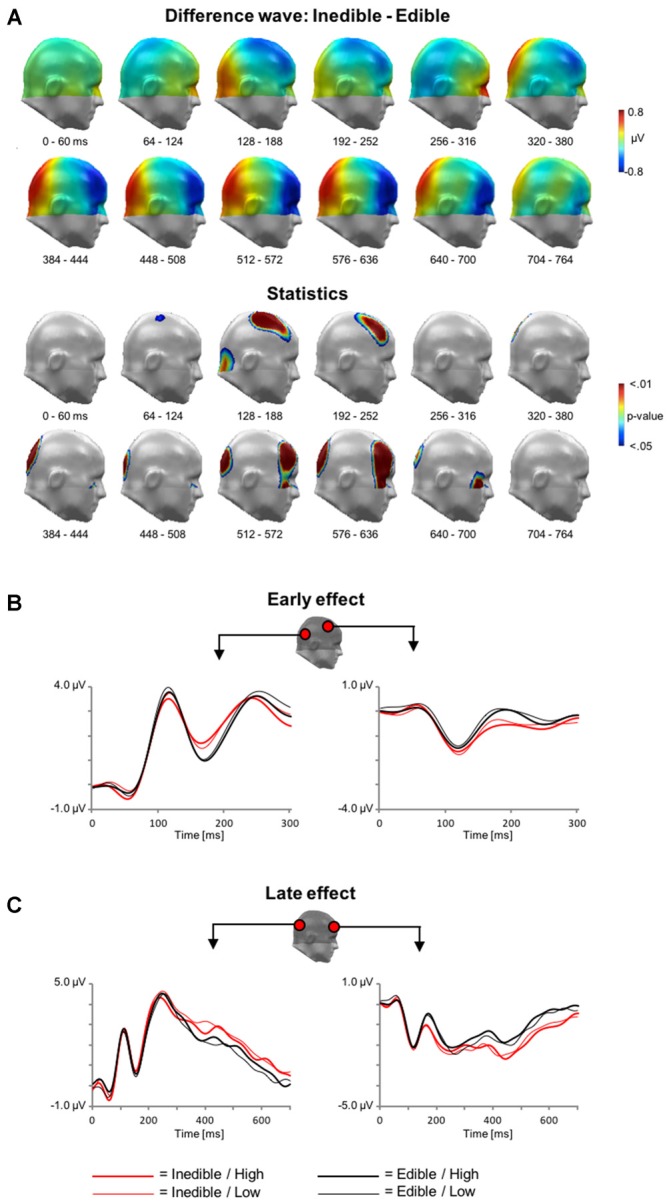
**Main effect of *Edibility* during electroencephalography (EEG) acquisition. (A)** Illustration of the topographical distribution of the scalp difference potentials (Inedible−Edible; top panel) and significant sensor cluster locations (*p* < 0.05–0.01; bottom panel) in 64 ms intervals from 0 to 764 ms. **(B)** Illustration of event related potential (ERP) waveforms for early effects (0–300 ms) in representative sensors over right lateral temporo-occipital and right central sensor sites. **(C)** Illustration of the late positive potential (LPP) waveform during later time windows (0–700 ms) for representative sensors over medial occipital and right frontal sensor sites.

Analogous to the analysis of the fMRI data, the first ERP analysis served to identify effects associated with the processing of inedible vs. edible food stimuli. The main findings are summarized in Figure 4 showing the scalp potential difference maps [Inedible−Edible], the statistics maps, as well as selected ERP waveforms of representative sensors within significant clusters.

In a time interval between ~100–300 ms, inspection of the scalp difference maps revealed that the processing of inedible food stimuli was associated with a relative positivity over inferior occipital sensor sites and an associated polarity reversal over fronto-central sensor sites, i.e., a relative negative potential for inedible food items. Statistical analysis indicated that the effect spanning the P1 and N1 wave was significant for the fronto-central sensor cluster, i.e., from 92 to 232 ms. Over parieto-occipital sensor sites, only the later effect covering the N1 wave reached significance, i.e., between 160–212 ms.

Subsequently, a topographically distinct effect emerged in a time interval between 300 and 1000 ms. Inspection of the scalp maps indicated that the processing of inedible foods was associated with an increased positivity over parieto-occipital sensor sites. The significance of this LPP effect was revealed by two sensor clusters spanning the time interval from 316 to 496 ms and 508–716 ms, respectively. Anterior sensor sites revealed a polarity reversal, i.e., a relative negative potential associated with the processing of inedible as compared to edible food items. Three distinct clusters from 336 to 740 ms, 528–728 ms, and 492–728 ms over left and right anterior clusters reached significance.

In a second step, the interaction of *Edibility by Intensity* was explored. However, no statistically significant effects were revealed in this analysis.

#### Main Effect of Intensity

**Figure 5:**
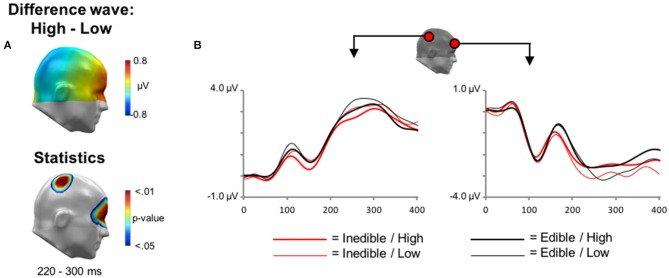
**Main effect of *Intensity* during EEG acquisition. (A)** Illustration of the topographical distribution of the scalp difference potentials (High−Low; top panel) and significant sensor cluster locations (*p* < 0.05–0.01; bottom panel) in a time window from 220 to 300 ms. **(B)** Illustration of ERP waveforms for representative sensors over right parietal and anterior frontal sensor sites.

Finally, main effects associated with the factor *Intensity* were explored. In the time interval between 200 and 300 ms, the processing of high intensity stimuli was associated with a relative negativity over central sensor sites and an associated polarity reversal over anterior regions, i.e., a relative positivity (see Figure 5). Statistical analysis indicated that this effect reached significance from 228 to 276 ms over central sensor regions and between 240–292 ms over anterior sites.

## Discussion

The consumption of food provides nutrients needed for survival, growth, and reproduction. However, the benefits of food intake need to be evaluated against costs associated with the intake of substances endangering health. Sensitivity to visual cues of natural decay of otherwise acceptable foods is critical for the detection of contaminated foods. The present data demonstrate the brain’s sensitivity to visual cues of foods that are spoiled or rotten.

It is a consistent finding in human neuroscience that salient and emotionally significant stimuli are discriminated early in the processing stream. For instance, emotional scenes, facial expressions, gestures, words and clashing moral statements are discriminated from neutral control items already around 150–200 ms after stimulus onset (Schupp et al., 2004; Kissler et al., 2006; Flaisch et al., 2009, 2011; Mühlberger et al., 2009; Van Berkum et al., 2009; Wieser et al., 2010; Flaisch and Schupp, 2013). Similar findings were observed in the food domain varying the appetitive value of food stimuli (Toepel et al., 2009; Meule et al., 2013). The present findings are consistent with these observations by demonstrating that the brain selectively responds to cues of natural decay early in the processing stream. Specifically, over posterior sensor sites, inedible and edible food items were discriminated between 150 and 200 ms, providing an upper bound for the time needed to extract information whether food is edible or inedible. Interestingly, the effect was already apparent around 100 ms post-stimulus, covering the P1 wave, which may relate to the detection of coarse visual characteristics of spoiled food items. As conscious stimulus representation is presumed to depend on several 100 ms of processing time (Chun and Potter, 1995), the differential brain responses to inedible and edible food stimuli occur too early to be based on deliberate (conscious) reasoning (Neely, 1977). Overall, the differentiation of edible and inedible food items early in the processing stream provides compelling evidence for the brain’s sensitivity to respond to visual cues associated with high risk food stimuli.

While the latency of the effect relates to the speed of the processing, the polarity of the effect is informative for delineating its functional significance. In the present study, the N1 wave was larger for edible as compared to inedible food items. While it seems tempting to relate the amplitude of the N1 component as an early reflection of the edibility dimension, a reduced N1 wave was observed for high-calorie as compared to low-calorie food stimuli in previous research (Toepel et al., 2009; Meule et al., 2013). Accordingly, the evidence that cues facilitating intake and cues associated with danger can elicit decreased N1 components is inconsistent with the notion that the N1 relates in straightforward manner to the acceptance or rejection of food stimuli. Instead, the data suggest that the N1 wave is modulated by the significance of the stimuli irrespective of the behavioral approach or avoidance response. Functionally, tagging sensory cues of foods related to the nutritional value or danger of foods seems a highly functional mechanism to regulate food intake.

Selecting cues of decay already at early perceptual stages appears advantageous for an organism’s survival, well-being, and reproduction, and was suggested to provide a basis for color preference and liking (Palmer and Schloss, 2010). However, according ERP-modulations may not exclusively indicate semantic-evaluative processes but could instead be triggered by purely physical differences between stimulus categories that either affect perceptual processes or interact with the semantic evaluation of according stimuli. Accordingly, the present study strived to increase the comparability of the stimulus materials with regard to a number of critical physical parameters, including figure-ground picture composition, lightning, and object distance (c.f., Meule et al., 2013). Conceivably, remaining physical stimulus differences may thus reflect systematic variations in diagnostic features such as color (mold shifts from white-to-green-to-black), brightness (i.e., a decrease over time) or size of food (i.e., shrinking over time) which are all cues associated with the natural decay of food items. However, the present study is not conclusive towards this end and additional research is needed to determine whether these physical stimulus characteristics are sufficient to elicit the observed ERP effects independent of their semantic implications.

The ERP data also revealed that spoiled and rotten foods modulate later stages of processing. Specifically, inedible food stimuli elicited a sustained enhanced positivity over parieto-occipital sensor sites, most pronounced between 500 and 650 ms after stimulus onset. Previous research relates the modulation of LPPs to the allocation of attentional resources (e.g., Stockburger et al., 2009; Svaldi et al., 2010; Blechert et al., 2014). Specifically, larger LPPs have been observed in vegetarians as compared to omnivores exposed to pictures of meat dishes (Stockburger et al., 2009), high-fat as compared to low-fat food stimuli (Toepel et al., 2009), liked vs. disliked food items (Harris et al., 2013), and in a binge eating as compared to overweight healthy control group exposed to high-calorie food stimuli (Svaldi et al., 2010). Despite some variation in terms of latency and topography, increased LPP amplitudes are presumed to reflect increased attention devoted to the processing of food stimuli. From this perspective, the present findings suggest that rotten and spoiled foods are more potent to draw attentional resources than edible and appetizing foods.

An attentional interpretation of the ERP data is strengthened by the functional imaging data. The processing of food pictures was enhanced in the extra-striate cortex with inedible foods showing an increased BOLD activation as compared to edible foods. Previous studies primarily focused on appetite and variables modulating food intake. Specifically, high-fat, liked, and appetizing food stimuli were contrasted to the processing of control food items. The most consistent findings of these studies regard structures in the extended visual cortex, with high incentive stimuli being associated with increased activation (Killgore et al., 2003; Beaver et al., 2006; Cornier et al., 2007; Goldstone et al., 2009; Passamonti et al., 2009; Born et al., 2011). The present findings contribute to the perspective that attention is selectively increased to dangerous food items in the extrastriate cortex. The observed pattern of findings could be seen as a specific instance of the general phenomenon that bad is stronger than good (Baumeister et al., 2001; Rozin and Royzman, 2001). Benefit and costs appear asymmetrical in that the intake of rotten and dangerous substances can cause serious harm to health, ordinarily outweighing the intake of nutrients. The difference in immediate consequences for well-being may also relate to differences in perceived arousal of the food stimuli, with spoiled and rotten food perceived as most arousing. Given the well-known phenomenon that hunger modulates the perception of food, it would be interesting to investigate in future studies how food deprivation affects the processing of contaminated foods (Schupp and Renner, 2011).

In contrast to extrastriate activation by spoiled foods, the processing of edible food items was associated with increased activation in the primary visual cortex. Given that significant effects emerged around 100 ms in the ERP study, the findings in the primary visual cortex reflect in all likelihood re-entrant processing rather than enhanced processing of initial stages of visual stimulus perception (Martínez et al., 1999).

Somewhat surprisingly, functional imaging data indicated no effects of edibility in key motivational structures. For instance, some studies showed that the processing of highly appetitive food stimuli, i.e., sweets and high-calorie food pictures, elicited greater activations in the amygdala (Killgore et al., 2003; Goldstone et al., 2009; Passamonti et al., 2009). Here, no differential activation for edible and inedible foods was observed in the amygdala, even when explored with relaxed statistical thresholds (SVC). However, findings regarding the amygdala show considerable variation across studies (Schupp and Renner, 2011; van der Laan et al., 2011) and the inclusion of high appetitive food stimuli may be needed to observe the effect in the absence of hunger (Goldstone et al., 2009). Furthermore, previous research observed increased activations in the anterior insular cortex when processing disgusting pictures (e.g., Beaver et al., 2006; Schienle et al., 2006; Calder et al., 2007; Stark et al., 2007). Again, even with relaxed statistical thresholding (SVC), there were no differential activations for edible and inedible food stimuli in subregions of the insular cortex in the present study. According to the saliency hypothesis (Menon and Uddin, 2010), inedible and edible food stimuli may both represent salient stimuli which would explain similar activations in the anterior insula. Engagement of the anterior insula, a key structure of the saliency network, is presumed to regulate attention processes (Menon and Uddin, 2010). However, there were pronounced differences in attentive processing of inedible and edible stimuli revealed by both, fMRI and ERP measures. To reconcile these somewhat inconsistent findings, the systematic variation of body state from deprivation to neutral hunger state to satiation seems most promising. Deprivation increases the incentive value of need-relevant stimuli and led to increases in the processing of need-related stimuli in distributed regions of the motive circuitry, i.e., aMCC, PCC, anterior insular cortex, and amygdala (LaBar et al., 2001; Mohanty et al., 2008; Goldstone et al., 2009; Becker et al., 2015). Furthermore, ERP studies revealed that deprivation affected the processing of appetitive food stimuli presumed to reflect increased attention to these stimuli when deprived (Stockburger et al., 2008). Conversely, over-consumption provides a mirror image on deprivation, rendering food inacceptable and decreasing activity in motivational regions (e.g., Small et al., 2001). Overall, to further the understanding of the activation of motivational regions by edible and inedible food stimuli, and to reveal the interaction with body state, future studies should systematically vary the hunger level from deprivation to satiation (see also Morrison and Salzman, 2010).

Natural decay provides a useful model system for the basic categorization of edibility of food items: seeing, smelling and tasting spoiled and rotten foods elicit a disgust response and strong behavior avoidance of potentially dangerous substances (Rozin and Fallon, 1987; Oaten et al., 2009; Schaller and Park, 2011; Tybur et al., 2013). Given that the only difference among the stimulus categories is associated with the changes of the food over time, variables leading to ambiguity in interpretation of the data were controlled, i.e., socio-cultural attitudes, composition of energy-density, and beliefs and thoughts about healthiness of the food product. However, it needs to be recognized that natural decay does not invariably lead to food rejection. A process of food enculturation can turn food items such as Gorgonzola and Roquefort cheese into delicacies. Obviously, this was not the case here, as participants showed a strong rejection response to the presented spoiled foods.

Prima fascia, the present findings support an evolutionary account of the processing of rotten and spoiled food items. Food stimuli show a similar neural signature in the present study to stimuli related to the fear and reproduction behavior system in terms of speed of processing and capture of attentional resources. One may accordingly posit that humans are evolutionarily prepared to signs of natural food decay, similar to emotional facial expression or body posture (Ohman and Mineka, 2001; de Gelder, 2006). However, the efficient capture of visual attention by decayed foods could also build upon a more general form of preparedness. Similar to the present findings, previous research demonstrated that emotional hand gestures are processed rapidly and draw attentional resources (Flaisch et al., 2009, 2011; Flaisch and Schupp, 2013). However, symbolic gestures are unlikely to be specifically prepared. They are cultural products invented and transmitted socially. Similarly, it has been suggested that humans’ knowledge about foods is largely acquired by experience and socio-cultural learning (Rozin and Vollmecke, 1986; Rozin, 1990; Rozin et al., 1999). Overall, the present findings would be consistent with evolutionary explanations assuming specific preparedness to respond to sensory cues of natural decay as well as an account proposing a general predisposition to acquire knowledge about food stimuli by learning from others.

## Author Contributions

All listed authors contributed substantial work to the preparation of this study.

## Conflict of Interest Statement

The authors declare that the research was conducted in the absence of any commercial or financial relationships that could be construed as a potential conflict of interest.

## Footnotes

^1^Control analyses excluding vegetarians from the analyses revealed similar findings as the full sample.
